# Faster and lower dose imaging: evaluating adaptive, constant gantry velocity and angular separation in fast low-dose 4D cone beam CT imaging

**DOI:** 10.1002/mp.16585

**Published:** 2023-07-10

**Authors:** Benjamin K. F. Lau, Owen Dillon, Shalini K. Vinod, Ricky T. O’Brien, Tess Reynolds

**Affiliations:** 1 Faculty of Medicine and Health, Image X Institute, University of Sydney, Sydney, NSW, Australia; 2 Liverpool & Macarthur Cancer Therapy Centres, Liverpool Hospital, Liverpool, New South Wales, Australia; 3 South Western Sydney Clinical School, The University of New South Wales & Ingham Institute for Applied Medical Research, Liverpool, New South Wales, Australia; 4 Medical Radiations, School of Health and Biomedical Sciences, RMIT University, Bundoora, Victoria, Australia

**Keywords:** 4DCBCT, angular separation, adaptive cone beam acquisition, gantry velocity, motion compensated reconstruction

## Abstract

**Background:**

The adoption of four-dimensional cone beam computed tomography (4DCBCT) for image-guided lung cancer radiotherapy is increasing, especially for hypofractionated treatments. However, the drawbacks of 4DCBCT include long scan times (∼240 s), inconsistent image quality, higher imaging dose than necessary, and streaking artifacts. With the emergence of linear accelerators that can acquire 4DCBCT scans in a short period of time (9.2 s) there is a need to examine the impact that these very fast gantry rotations have on 4DCBCT image quality.

**Purpose:**

This study investigates the impact of gantry velocity and angular separation between x-ray projections on image quality and its implication for fast low-dose 4DCBCT with emerging systems, such as the Varian Halcyon that provide fast gantry rotation and imaging. Large and uneven angular separation between x-ray projections is known to reduce 4DCBCT image quality through increased streaking artifacts. However, it is not known when angular separation starts degrading image quality. The study assesses the impact of constant and adaptive gantry velocity and determines the level when angular gaps impair image quality using state-of-the-art reconstruction methods.

**Methods:**

This study considers fast low-dose 4DCBCT acquisitions (60–80 s, 200-projection scans). To assess the impact of adaptive gantry rotations, the angular position of x-ray projections from adaptive 4DCBCT acquisitions from a 30-patient clinical trial were analyzed (referred to as patient angular gaps). To assess the impact of angular gaps, variable and static angular gaps (20°, 30°, 40°) were introduced into evenly separated 200 projections (ideal angular separation). To simulate fast gantry rotations, which are on emerging linacs, constant gantry velocity acquisitions (9.2 s, 60 s, 120 s, 240 s) were simulated by sampling x-ray projections at constant intervals using the patient breathing traces from the ADAPT clinical trial (ACTRN12618001440213). The 4D Extended Cardiac-Torso (XCAT) digital phantom was used to simulate projections to remove patient-specific image quality variables.

Image reconstruction was performed using Feldkamp-Davis-Kress (FDK), McKinnon-Bates (MKB), and Motion-Compensated-MKB (MCMKB) algorithms. Image quality was assessed using Structural Similarity-Index-Measure (SSIM), Contrast-to-Noise-Ratio (CNR), Signal-to-Noise-Ratio (SNR), Tissue-Interface-Width-Diaphragm (TIW-D), and Tissue-Interface-Width-Tumor (TIW-T).

**Results:**

Patient angular gaps and variable angular gap reconstructions produced similar results to ideal angular separation reconstructions, while static angular gap reconstructions produced lower image quality metrics. For MCMKB-reconstructions, average patient angular gaps produced SSIM-0.98, CNR-13.6, SNR-34.8, TIW-D-1.5 mm, and TIW-T-2.0 mm, static angular gap 40° produced SSIM-0.92, CNR-6.8, SNR-6.7, TIW-D-5.7 mm, and TIW-T-5.9 mm and ideal produced SSIM-1.00, CNR-13.6, SNR-34.8, TIW-D-1.5 mm, and TIW-T-2.0 mm. All constant gantry velocity reconstructions produced lower image quality metrics than ideal angular separation reconstructions regardless of the acquisition time. Motion compensated reconstruction (MCMKB) produced the highest contrast images with low streaking artifacts.

**Conclusion:**

Very fast 4DCBCT scans can be acquired provided that the entire scan range is adaptively sampled, and motion-compensated reconstruction is performed. Importantly, the angular separation between x-ray projections within each individual respiratory bin had minimal effect on the image quality of fast low-dose 4DCBCT imaging. The results will assist the development of future 4DCBCT acquisition protocols that can now be achieved in very short time frames with emerging linear accelerators.

## 1 | INTRODUCTION

Radiotherapy is a prominent treatment option for lung cancer, which is the leading cause of cancer-related deaths worldwide.^1^ Radiotherapy relies on high-precision imaging to ensure the safe delivery of radiation to cancer while sparing surrounding healthy tissue. Multiple image modalities are utilized throughout the various stages of a patient’s radiotherapy treatment from treatment planning to pre-treatment patient alignment as well as imaging during the treatment delivery. Focusing on pre-treatment imaging for patient alignment, 4D Cone Beam Computed Tomography (4DCBCT) is becoming an indispensable tool utilizing the on-board kV imager found on most linear accelerator gantries.^2–4^ Notably, it has assisted in facilitating short-duration stereotactic dose-escalation radiotherapy techniques for lung cancer patients.^2,3,5^

Despite the success of conventional 4DCBCT imaging, there remain limitations to the modality.^6,7^ Efforts to improve conventionally or develop alternative 4DCBCT imaging are driven by the possibility of reducing imaging dose, thereby reducing the risk of secondary cancers and allowing for more frequent imaging. Shorter scan times with lower doses are expected to further increase the precision of treatment, reduce scan, improve patient comfort and increase patient throughput through the shorter scan times.

Numerous methods to reduce imaging dose and scan time during 4DCBCT imaging have previously been proposed, commonly employing novel reconstruction algorithms (e.g., iterative and motion compensated^8–11^). A prevailing disadvantage that currently plagues many reconstruction algorithms is the computational time, with promising techniques ranging from under 10 min^12–14^ through to several hours.^8,9,15–17^ Modern Graphics Processing Units are assisting in reducing computation time; however, some algorithms still require long computational times preventing transition to the clinic. An additional barrier to overcome in a clinical setting is removing the need for manual intervention (i.e., segmentation of the lung volume) that forms part of the process for some image reconstruction algorithms.^18–20^

In addition to developing specialized reconstruction algorithms, adapting the gantry velocity and projection time interval between projections has been investigated as a pathway to reducing 4DCBCT imaging dose and scan time. One such example is adaptive 4DCBCT^21,22^ (referred to in some literature as respiratory motion guided 4DCBCT or RMG-4DCBCT^23–27^), which monitors a patient’s breathing signal and modulates gantry velocity and projection time interval in real time. Combining adaptive 4DCBCT with motion-compensated reconstruction algorithms has enabled imaging dose to be reduced by 85% and scan time to be reduced by up to 75%.^22^ Most recently, the feasibility of implementing adaptive 4DCBCT techniques in a clinical setting, has been examined in the Adaptive CT Acquisition for Personalized Thoracic imaging (ADAPT) clinical trial (ACTRN12618001440213).^21,22,28^

A pillar for the success of adaptive 4DCBCT is an even angular separation of projections across all respiratory phases. However, this is not always trivial to achieve. For instance, in fast acquisitions with a constant velocity and project time interval, irregularities in breathing characteristics (e.g., period and displacement) can lead to an uneven angular distribution of projections within each respiratory phase bin.^29,30^ Similarly, for variable velocity and projection time interval acquisitions, mechanical constraints and velocity limitations enforced by the International Electrotechnical Commission (IEC) may restrict the complexity and frequency of velocity changes, which can result in a deviation from the desired even angular distribution of projections within each respiratory phase bin.^31^

With the emergence of new linear accelerators, such as the Halcyon from Varian Medical Systems, that are capable of acquiring 3D datasets in as little as 9.2 s, there is a need to determine if these very fast gantry rotations are able to achieve suitable projection spacing across all 10 respiratory phases required to reconstruct a 4DCBCT image. To achieve this aim, this study evaluated the impact of gantry velocity (constant and adaptive) and angular separation between x-ray projections on image quality in the context of fast low-dose 4DCBCT acquisitions and emerging reconstruction methods. The study determined the level at which angular gaps impair image quality and investigated whether constant and adaptive gantry velocity acquisitions are viable with state-of-the-art linear accelerators and state-of-the-art reconstruction methods. The key innovation in this study is identifying system requirements (i.e., the precision of gantry speed control) for adaptive acquisitions and the impact of image quality for very fast rotating gantries. The results of this study will be significant and of interest to the CBCT research community, considering the increasing presence of faster-rotating gantry systems in the radiation therapy market. All in all, this study will provide a guide for the future development of 4DCBCT acquisition protocols on the newest generation of linear accelerators.

## 2 | MATERIALS AND METHODS

**FIGURE 1:**
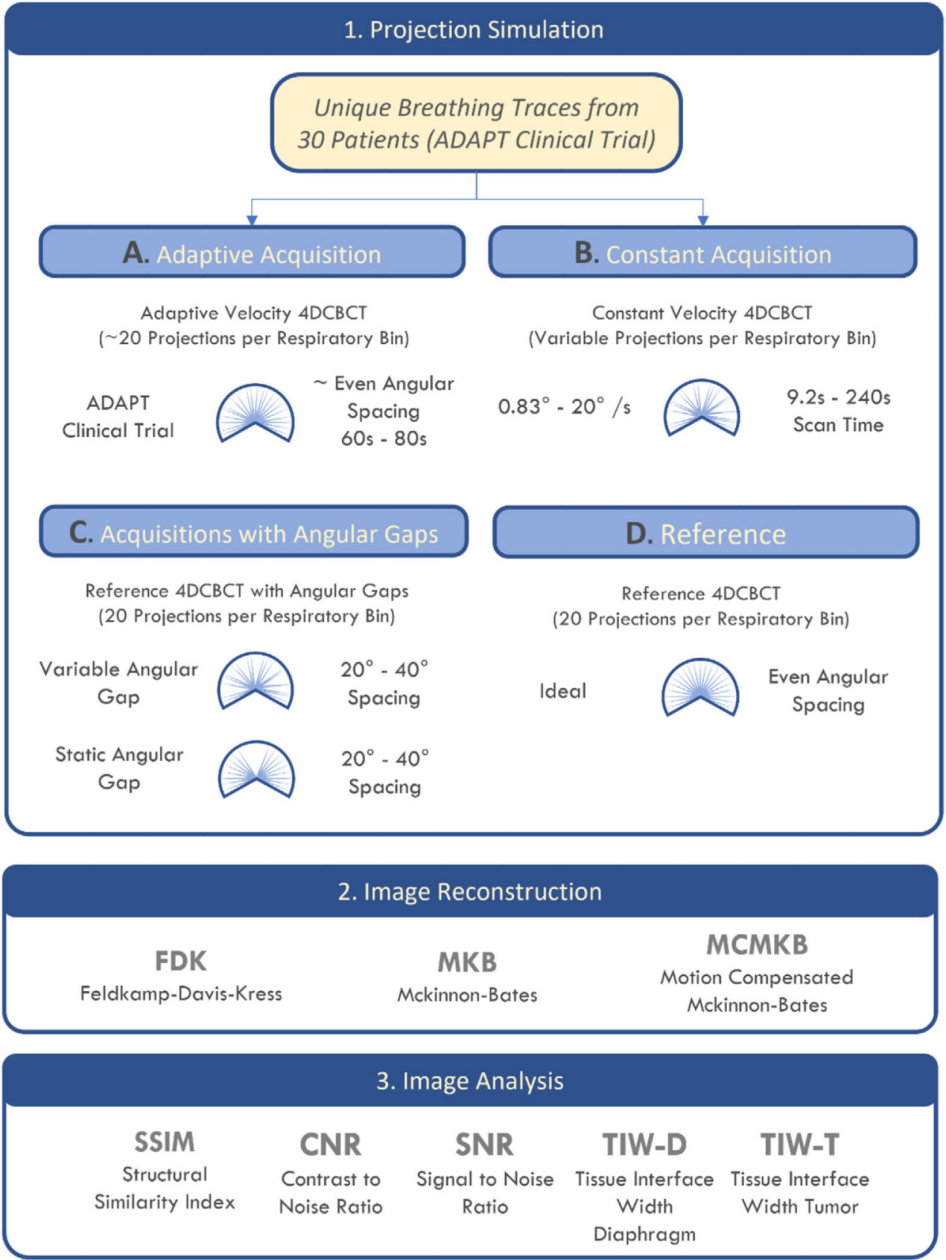
Summary of the study workflow. Patient and simulated respiratory data were separated into 10 respiratory phases and a XCAT volume was generated for each phase. Image reconstruction was performed using FDK, MKB, and MCMKB algorithms and images were analyzed using SSIM, CNR, SNR, TIW-D, and TIW-T.

The study schematic is detailed in Figure 1. Fast low-dose adaptive 4DCBCT acquisition data and simulated angles were projected through a 4D extended cardiactorso (XCAT) digital phantom^32^ to simulate 4DCBCT acquisitions. Fast low-dose adaptive (200 projections in 60−80 s) 4DCBCT acquisition data (respiratory phase bin and angular position of each projection acquired) from all 30 patients from the ADAPT clinical trial (see next section) were analyzed, identifying the average angular separation, maximum angular separation, and root mean square error (RMSE) of the angular separation between x-ray projections from each scan. Reconstructions were generated using Feldkamp–Davis-Kress (FDK),^33^ McKinnon-Bates (MKB),^34^ and Motion Compensated McKinnon Bates (MCMKB)^28^ algorithms and then analyzed using a range of image quality metrics.

### 2.1 | Patient breathing data used to simulate acquisition: The ADAPT clinical trial

The aim of the ADAPT clinical trial was to eliminate redundant projections that are inherent during 4DCBCT image acquisition and to also reduce streaking artifacts as a result of irregular breathing by synchronizing the gantry rotation speed and projection pulse rate to real-time changes in the patient’s breathing rate. For example, if the patient breathes faster, the gantry was rotated faster. Similarly, if the patient’s breathing slows then so does the gantry rotation speed.

Legal, ethics, and regulatory compliance were obtained for the ADAPT clinical trial (Australia New Zealand clinical trial registry number ACTRN12618 001440213). All 30 patients from the ADAPT clinical trial were examined in this study.

The ADAPT clinical trial focused on pre-treatment 4DCBCT for lung cancer radiotherapy.^21^ For two treatment fractions delivered, the patient cohort received a conventional 1320 project, 4 min, 4DCBCT scan for alignment and radiotherapy treatment as per routine standard of care. After the completion of the radiotherapy treatment, the patient cohort immediately received two additional adaptive 4DCBCT scans. The adaptive scans were characterized as either being fast low-dose (200 projections in approximately 60−80 s) or standard (600 projections in 240 s) scans. Adaptive 4DCBCT scans were only acquired on the first two fractions across two separate days. For this simulation study, we focus only on the fast low-dose adaptive scans (200 projections in approximately 60−80 s).

An Elekta Synergy linear accelerator (Elekta AB, Sweden) was used to provide treatment and acquisition. The Elekta Synergy makes use of fixed kV/mA per projection and was set to acquire scans at 120 kV, 20 mA, and 25 ms (0.5mAs per projection).

The adaptive acquisition in the ADAPT clinical trial used adaptive acquisition to modulate the gantry velocity and projection rate (through projection suppression) of a linear accelerator due to variations in the patient’s breathing. Details of the underlying mathematical optimization techniques, the electronics circuits, and real-time gantry control of the linear accelerator used during an adaptive acquisition are detailed elsewhere.^35,36^

### 2.2 | Simulation study and digital volume generation for both adaptive and constant gantry speed acquisitions

The simulation of all scans was completed in four steps; (1) digital volume generation, (2) projection simulation, (3) image reconstruction, and (4) assessment (angular separation and image quality analysis). These steps are depicted in Figure 1 and detailed in the sections below.

To mimic realistic patient physiology and anatomy movement during image acquisition, the 4D XCAT digital phantom was used. The XCAT is a programmable phantom that enables time series volumes, derived from patient scans, to be generated with breathing characteristics (period and magnitude of displacement) defined by the user.^32^ As we are only concerned with investigating the role of the gantry velocity and angular separation on image quality, a sinusoidal breathing trace (period 4 s, displacement of 1 cm in the superior to inferior direction) was used to generate the XCAT phantom (whereas patient breathing traces were used for projection simulation). A spherical tumor with a diameter of 2 cm and displacement of 1 cm from the superior to inferior direction was placed in the lung of the phantom. The breathing trace was separated into 10 respiratory phases and a XCAT volume was generated for each respiratory phase that is used for projection simulation.

### 2.3 | Projection simulation—constant velocity acquisition

#### 2.3.1 | Constant velocity acquisition

Patient respiratory phase data from the ADAPT clinical trial were used to simulate patient scans on constant velocity gantry systems. Respiratory phases were sampled at constant intervals (as opposed to variable intervals in adaptive 4DCBCT) throughout a 200° arc to simulate constant velocity acquisitions. To study the effects of gantry rotation speed against image quality, four scenarios were simulated to be inclusive of the fastest possible gantry rotation speed (Varian Halcyon 200° rotation in 9.2 s) to the current conventional standard (Elekta Synergy 200° in 240 s).

These scenarios include 9.2, 60, 120, and 240 s (Figure 1b). The 9.2 s (20°/s) scenario represents an 200° arc acquisition on the Varian Halcyon which is capable of rotating at 360° in 16.6 s. The 60 s (3.3°/s) scenario represents a similar time required for a 200-projection adaptive 4DCBCT scan or a 3DCBCT. The 120 s (2.5°/s) scenario represents the same amount of time required for a 4DCBCT scan using the default settings on the Varian TrueBeam v2.7. The 240 s (0.8°/s) scenario represents the most common amount of time required for a conventional 4DCBCT scan.^6,30^

As adaptive 4DCBCT scans ranged from 60 to 80 s, for constant velocity simulations that exceeded this threshold (120 and 240 s) patient breathing traces were extrapolated by looping the traces to ensure 200 projections could be sampled at a constant interval.

### 2.4 | Projection simulation—Acquisitions with angular gaps

To better understand the impact of angular gaps in the project space, a series of simulated acquisitions were performed using variable and static angular gaps. The aim of this part of the study was to determine the angular gap limits where image quality is no longer suitable for clinical use.

#### 2.4.1 | Ideal angular gap

As a point of reference, the Ideal angular gap simulation (Figure 1d) was generated by assuming that the gap between consecutive projections in each respiratory phase bin are uniformly distributed (i.e., the angular gaps between each projection are equal). This simulation is not achievable in practice unless the patient has perfectly regular breathing, but it will provide a reference point on the best image quality possible for a given number of projections.

Evenly spaced low-dose 200 projection adaptive 4DCBCT (1 projection acquisition per 1° of gantry rotation) scans were simulated. Twenty projections were allocated for each of the 10 respiratory phases, with an angular separation of 10°, respectively. That is, Bin 1 (0°, 10°, 20°, 30°…180°, 190°), Bin 2 (1°, 11°, 21°, 31°… 181°, 191°), and so forth. These scans were used as the baseline ideal for all scenarios.

#### 2.4.2 | Variable angular gap

Variable angular gaps (Figure 1c) simulate the rapid transition through a respiratory phase due to fast breathing or rapidly changing irregular breathing, this causes the acquisition to miss a respiratory bin. A variable angular gap of 20°, 30°, and 40° was introduced to the ideal projection locations within each respiratory phase bin, the location of the angular gap was varied across the respiratory bins. The variable angular gap scenario maintains the correct number of projections (200) with 20 projections in each respiratory bin across the 200° scan range, however, projections are no longer evenly spaced.

#### 2.4.3 | Static angular gap

The static angular gap (Figure 1c) simulates a patient holding their breath during the acquisition, and no projection acquisition occurred during the long pause for the same continuous gap across all respiratory phases. In addition, a static angular gap of 20°, 30°, and 40° was introduced to the ideal projection locations, however, the angular gap location was not varied throughout all respiratory bins but was kept at the same location, to simulate no data acquisition for the same region across all respiratory phases. Currently, this is a limitation of the adaptive acquisition protocol implemented in the ADAPT clinical trial because static angular gaps may occur as the imaging gantry cannot stop completely during large breathing pauses.

### 2.5 | Projection simulation—Adaptive velocity acquisition

#### 2.5.1 | ADAPT clinical trial—Adaptive acquisition data

The adaptive acquisitions from the ADAPT clinical trial provided unique breathing traces for 30 patients (first two treatment fractions). The breathing traces provide the exact angular position of each projection acquired along with its associated respiratory phase bin. Using this information projections in the same locations can be simulated through the XCAT software phantom and then allocated into the corresponding respiratory bins to reflect a realistic acquisition (Figure 1a).

### 2.6 | Image reconstruction

Following the projection simulation for all acquisitions, three different image reconstruction methods were utilized to generate the final image volumes. Detailed mathematical summaries of the algorithms used in this study can be found here.^22^

#### 2.6.1 | Feldkamp-Davis-Kress (FDK)

The 4DFDK method is the current clinical standard for 4DCBCT reconstructions.^33^ All 4DFDK reconstructions were reconstructed using the RTK implementation of FDK. Sinogram padding of 10 pixels and 0.9 Hann filtering coefficient was applied to reconstruct images similar to those in current commercial clinical systems.

#### 2.6.2 | McKinnon-Bates (MKB)

The MKB method also relies on filtered back projection, utilizing 4DFDK volumes and simulated 4DFDK volumes (generated from 3DFDK volume) to reconstruct MKB volumes.^34^ The intention of the MKB method is to reconstruct volumes that resemble low motion blur 4DFDK volumes and low noise 3DFDK volumes. The MKB implementation in this study makes use of difference volumes rather than difference projections as described in the original MKB article to prevent overflow problems.

#### 2.6.3 | Motion compensated Mckinnon-Bates (MCMKB)

The MCMKB method accounts for respiratory motion by back projecting along curved paths.^28^ MKB respiratory binned volumes undergo Deformable Image Registration (DIR) to generate Deformable Vector Fields (DVFs). These are then applied to the original MKB volumes to MCMKB volumes.

### 2.7 | Image reconstruction hardware and settings

The hardware specifications for the workstation used was a Nvidia GPU, RTX 8000 (48GB VRAM and 4608 CUDA cores), Intel Xeon E5–2687 with 32 logical cores at 3.1 GHz CPU and 64GB of RAM. All Reconstruction algorithm implementations were performed using Reconstruction Tool kit (RTK). GPU acceleration was used for all reconstruction algorithms. DIR was performed using the Elastix Toolkit. All images were reconstructed to 300 × 300 × 300 voxels, where each voxel is 1 mm^3^.

### 2.8 | Image quality metrics

Image quality of all reconstructions were quantified using Structural Similarity Index Measure (SSIM), Signal-to-Noise Ratio (SNR) and Contrast-to-Noise Ratio (CNR). The ideal angular separation reconstructions were used as the ground truth for SSIM computations. Tissue Interface Width Diaphragm (TIW-D) and Tissue Interface Width Tumor (TIW-T) was quantified as in Riblett et al.,^37^ with Region of Interest (ROI) voxels placed over the diaphragm edge and tumor region respectively. To avoid truncation artifacts, image quality metrics were calculated over ROI defined within the field of view.


$$SSIM=\frac{\left(2\mu_{gt}\mu_{r}+c_{1}\right)\left(2\sigma_{r,gt}+c_{2}\right)}{\left(\mu_{gt}^{2}+\mu_{r}^{2}+c_{2}\right)\left(\sigma_{gt}^{2}+\sigma_{r}^{2}+c_{2}\right)}$$

We denote μ and $\sigma^{2}$ as the voxel average and variances of ROI, $c_{1}={\left(0.01L\right)}^{2}$ and $c_{2}={\left(0.03L\right)}^{2}$ with L being the dynamic range of the volumes. We define the signal-to-noise ratio and contrast-to-noise ratio using:

$$SNR=\frac{\mu_{Diaphragm}}{\sigma_{Diaphragm}}$$

$$CNR=\frac{\left|\mu_{Diaphragm}-\mu_{Lung}\right|}{\sigma_{Diaphragm}}$$

where μ is the voxel value average, and σ is the standard deviation of the voxel values in the volume of interest. A higher SNR result is indicative of more signal than noise. A higher CNR result indicates better image contrast.

To quantify image sharpness, we use:

$$f(x,a,c)=\frac{1}{1+e^{-a(x-c)}}$$

$$\left(a_{j},c_{j}\right)=\underset{a,c}{\min}\left\{{\left\|V_{j}-f(x,a,c)\right\|}_{2}^{2}\right\}$$

$$TIS=\frac{1}{25}\sum_{j=1}^{25}a_{j}=\mu_{c}$$

TIW=2log(9)/TIS

TIW-D computes a 5 × 5 × l ROI across the diaphragm, where l is a run of voxels in the superior-to-inferior axis direction. TIW-D measures the width of the diaphragm edge. TIW-T computes a 5 × 5 × l ROI across the tumor, where l is a run of voxels in the superior-to-inferior axis direction. TIW-T measures the width of the tumor edge. A lower TIW result indicates a sharper edge.

## 3 | RESULTS

### 3.1 | The effect of constant velocity acquisition on image quality

**FIGURE 2:**
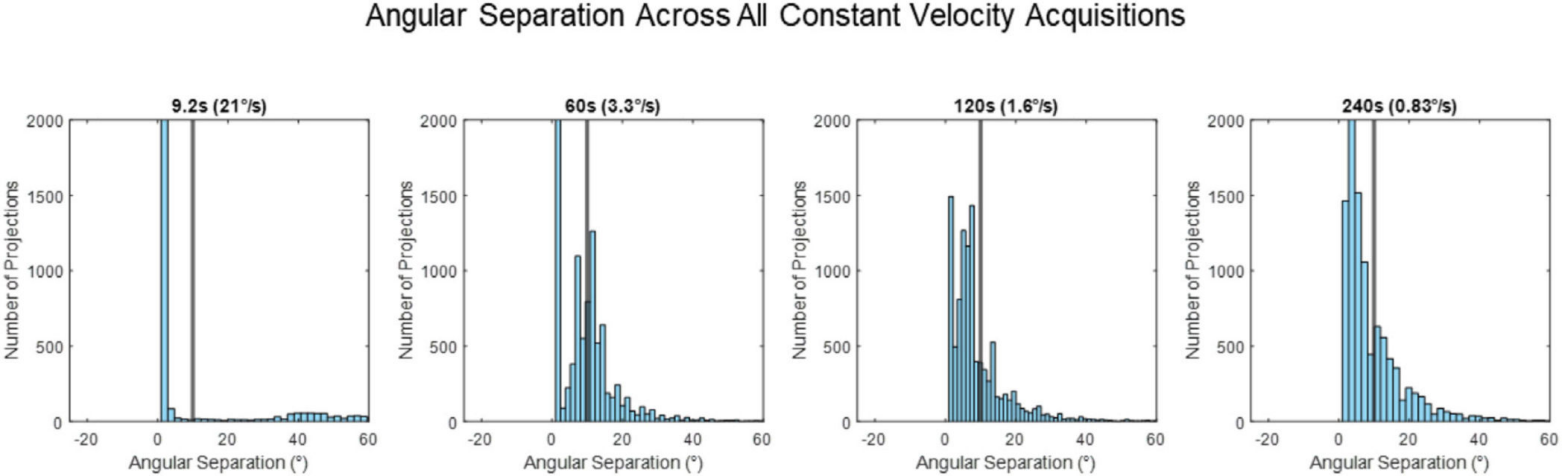
Histograms of the angular separation between x-ray projections observed across all the constant velocity acquisitions. The black line represents even 10° angular spacing, the ideal angular separation between x-ray projections.

**FIGURE 3:**
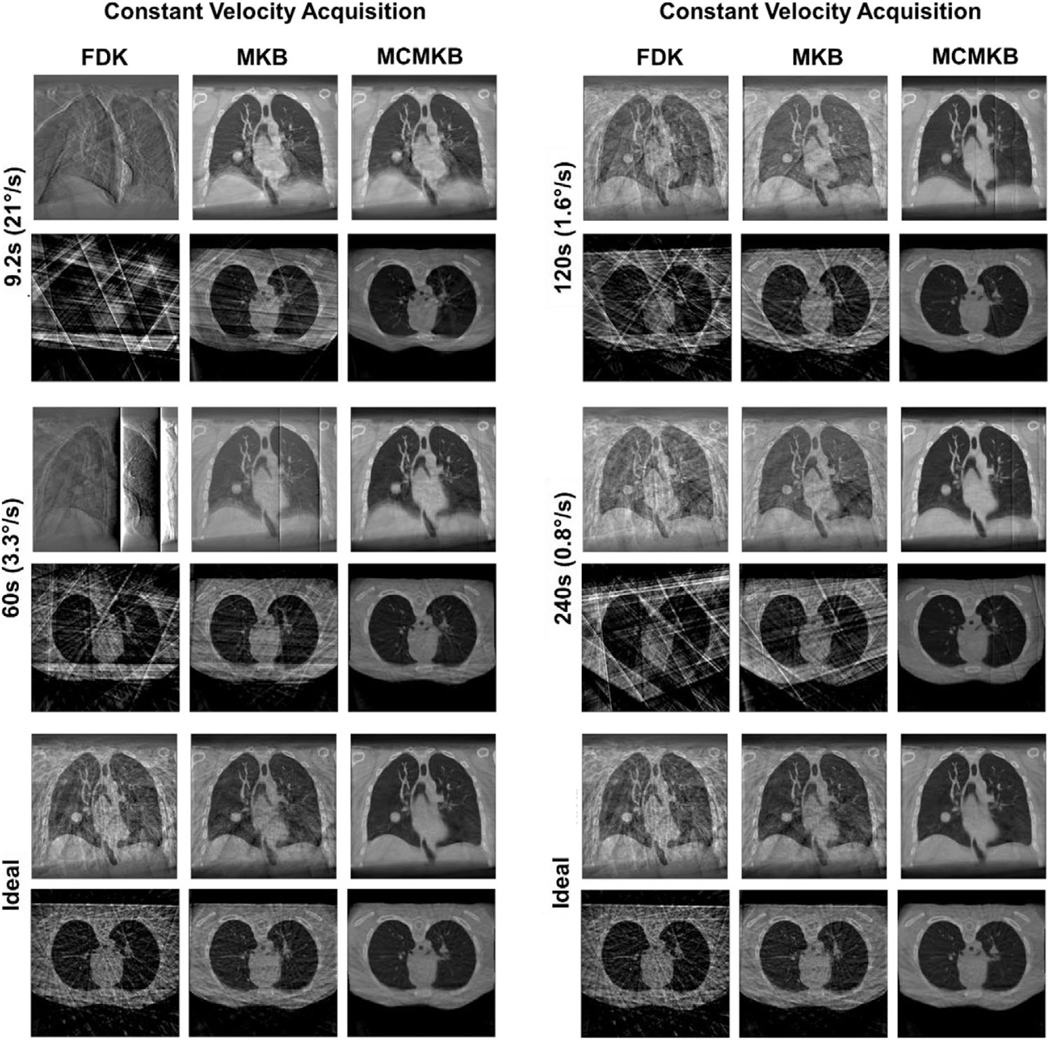
Reconstruction images for constant velocity acquisitions. Ideal angle separation image reconstructions are included for reference. FDK, MKB, and MCMKB reconstructions at peak exhale phase 1 are displayed.

Figure 2 provides histograms of the angular separation between x-ray projections for all the constant velocity acquisitions. Figure 3 provides images of the constant velocity acquisition reconstructions for a single patient fraction, this is reflective of the general pattern across the cohort of images studied in this paper.

#### 3.1.1 | Qualitative analysis (constant velocity acquisition)

Images in Figure 3 show that constant velocity acquisition reconstructions were noticeably streakier and contained more motion blur compared to ideal angular separation reconstructions FDK and MKB reconstructions were considerably streakier, nosier, and exhibited more motion blur compared to the ideal angular separation reconstructions. These visual observations were confirmed by quantitative metrics (see below). Faster 9.2 and 60 s acquisitions FDK and MKB reconstructions were streakier, nosier, and exhibited more motion blur compared to the ideal angular separation reconstructions, whereas slower 120 and 240 s acquisition FDK and MKB reconstructions were occasionally visually similar to ideal reconstructions in certain respiratory phases. MCMKB reconstructions reduced some streaks, noise, and motion blur that were present in FDK and MKB reconstructions. Faster 9.2 and 60 s acquisitions MCMKB reconstructions exhibited motion blur around the diaphragm and tumor, slower 120 and 240 s acquisition reconstructions reduced motion blur however streaks became more apparent.

The main reason for these observations is the inconsistent sampling of projections across each individual phase. Faster acquisitions had shorter breathing traces that increased the likelihood of under-sampling projections in certain respiratory phases, resulting in inconsistent image quality across all 10 respiratory phase correlated volumes. Although slower acquisitions enabled longer breathing traces, they did not ensure all respiratory phases were evenly sampled, as shown by the residual motion blur and streaking artifacts (120 and 240 s MCMKB reconstructions). Consequently, DVFs could not be accurately estimated for undersampled respiratory bins, and image artifacts propagated into the final MCMKB reconstructions. Another factor contributing to the reduced image quality of constant velocity acquisitions compared with the ideal scenario was the presence of large and small angular gaps, as observed in the histograms presented in (Table 1 and Figure 2). Across all rotational speeds, constant velocity acquisitions had large average angular separation, maximum angular separation, and angular RMSE well beyond the ideal angular separation (Table 1 and Figure 2).

#### 3.1.2 | Quantitative analysis (constant velocity acquisition)

In general, constant velocity acquisition reconstructions produced lower image quality metrics results than ideal angular separation for FDK, MKB, and MCMKB reconstructions (Tables 2–6). The qualitative analysis demonstrated that constant angular velocity reconstructions resulted in a reduction in visual image quality, and this was reflected in the quantitative metrics. For example, 9.2 s acquisition MCMKB reconstructions had SSIM 0.97 ± 0.00, CNR 13.1 ± 3.5, SNR 35.4 ± 12.7, TIW-D 5.8 ± 12.3 mm, TIW-T 5.3 ± 12.1 mm compared to ideal angular separation MCMKB reconstruction of SSIM 1.00, CNR 13.6, SNR 34.8, TIW-D 1.5 mm, and TIW-T 2.0 mm. Faster 9.2 and 60 s constant velocity acquisitions showed the greatest reduction in TIW values which was reflected in the qualitative analysis.

Although slower 120 and 240 s constant velocity acquisitions showed improvements across all image quality metrics, TIW results were still considerably reduced compared to the ideal reconstructions. The 240 s acquisition MCMKB reconstructions had SSIM 0.98 ± 0.01, CNR 16.7 ± 2.2, SNR 43.9 ± 10.9, TIW-D 4.1 ± 8.6 mm, TIW-T 3.6 ± 9.1 mm. TIW was also impacted in 240 s acquisitions FDK (TIW-D 3.2 ± 7.6 mm, TIW-T 3.5 ± 10.8 mm) and MKB (TIW-D 3.3 ± 22.4 mm, TIW-T 2.9 ± 14.3 mm) reconstructions. Furthermore, the standard deviation across all image quality metrics was higher for constant velocity acquisition reconstructions, making it difficult to produce consistent and predictable image quality with constant gantry speeds. Therefore, regardless of the gantry rotational speed in constant velocity acquisition (200-projections), it is likely image quality will be hindered by streaking or motion artifacts.

### 3.2 | The effect of angular gaps on image quality

**FIGURE 4:**
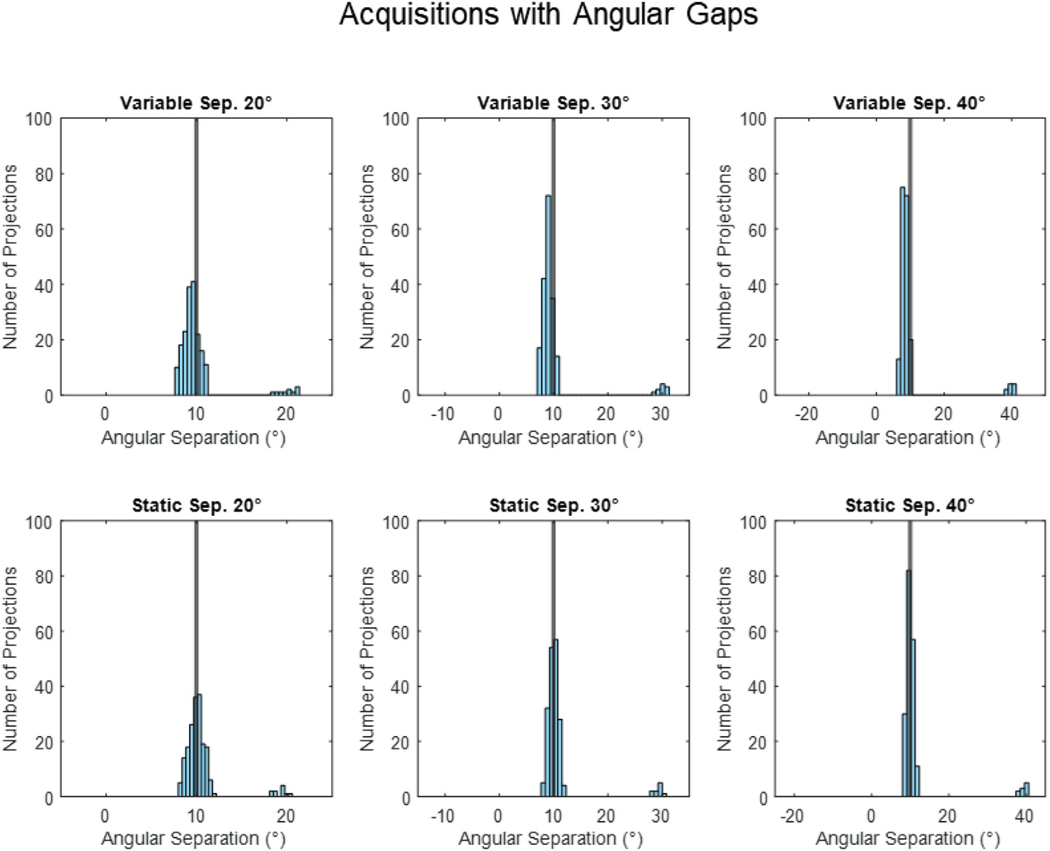
Histograms of the angular separation between x-ray projections observed in the variable and static angular gaps of 20°, 30°, and 40° acquisitions. The black line represents even 10° angular spacing, the ideal angular separation between x-ray projections.

**FIGURE 5:**
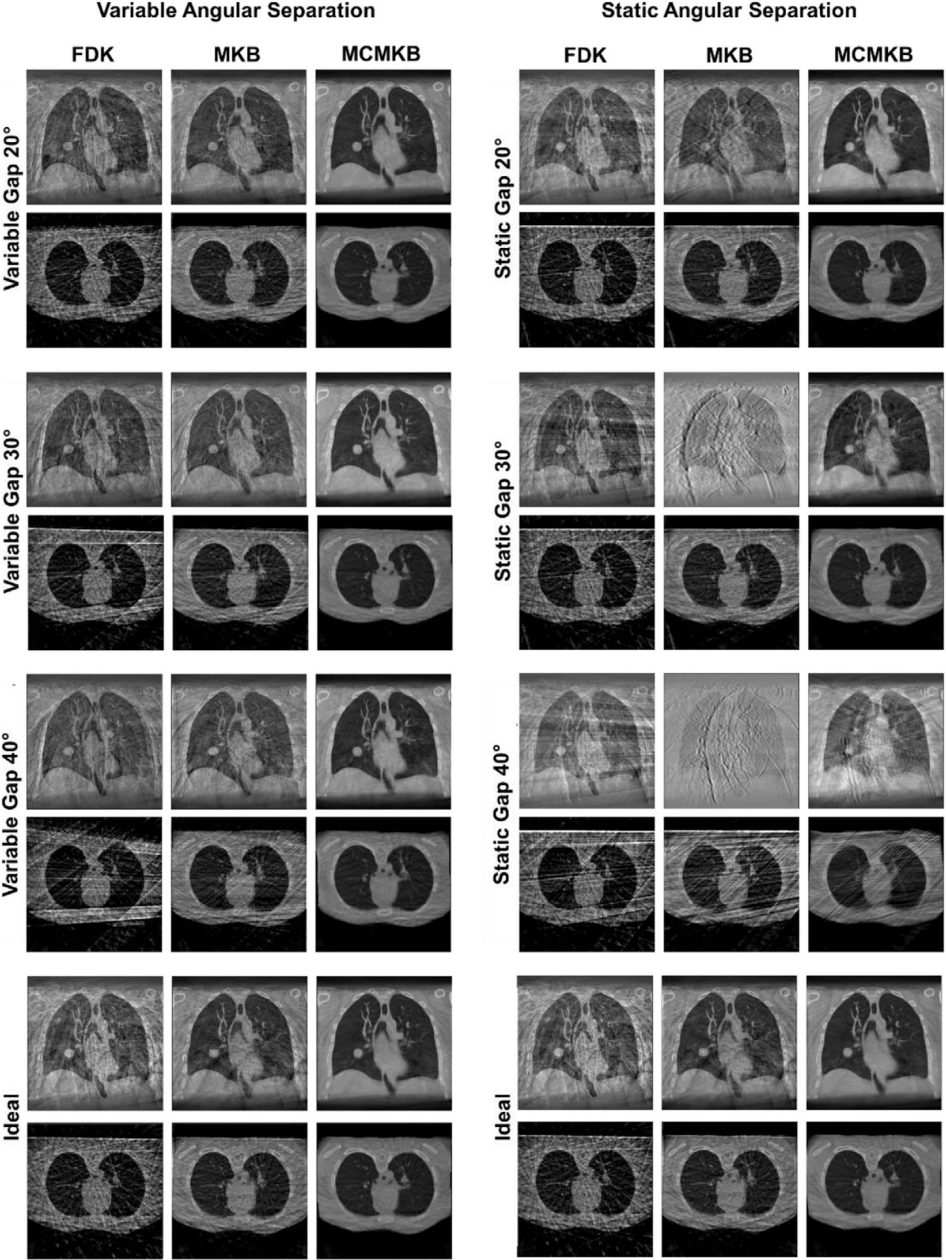
Reconstructed images for variable and static angular gaps of 20°, 30°, and 40°. Ideal angular separation image reconstructions are included for reference. The images comprise of FDK, MKB, and MCMKB reconstructions at peak exhale phase 1.

Figure 4 provides histograms of the angular separation between x-ray projections for all the variable and static velocity acquisitions. Figure 5 provides images of the variable and static velocity acquisition reconstructions.

For variable and static angular gaps, both scenarios had similar histogram distributions (Figure 4). The histograms were mostly distributed around the 10° region with a small number of projections with an angular gap at the simulated gaps, that is, 20°, 30°, and 40° regions.

For variable angular gap acquisitions, with separations of 20°, 30°, and 40° between one pair of adjacent projections occur at different positions within each respiratory bin, simulating rapid transitions between rapidly changing, irregular breathing. For static angular separation acquisitions, the angular separation between adjacent projections exists in the same angular position across all respiratory bins, simulating a long pause in breathing.

To assess the impact of both variable and static angular gaps in the acquired projections on image reconstruction, specific locations within the evenly separated (ideal) 4DCBCT acquisitions were modified. The majority of projections remained the same as in the ideal case (i.e., 10° separation), with the exception of the introduced modifications to simulate the static or variable angular gaps. This approach aimed to isolate and analyze the effects of these angular gaps on the reconstructed images.

While variable and static angular gap acquisitions have similar histogram profiles, both yield different reconstruction results. This highlights the importance of sampling the entire 200° angular range and will be further explored in the subsequent sections.

The greater the variable or static gap the higher the angular RMSE and maximum angular separation compared to the ideal angular separation (Table 1).

#### 3.2.1 | Variable angular gaps

The variable angular gap 20°, 30°, and 40° reconstructions were visually similar to the ideal reconstructions (Figure 5). FDK and MKB reconstructions were marginally streakier and nosier than ideal angular separation reconstructions. MCMKB reconstructions for variable angular gaps of 20°, 30°, and 40° had the least streaks and noise and closely resembled the ideal angular separation MCMKB reconstructions. Increasing the variable gap between projections did not have a noticeable effect on reconstruction image quality as the projections covered the entire scan range.

Variable angular gaps of 20°, 30°, and 40°FDK, MKB, and MCKMB reconstructions produced similar image quality metrics results compared to the ideal angular separation reconstructions (Tables 2–6). However, increasing the variable angular gap did reduce some image quality metric results. For example, the variable angular gap 20° with the MCMKB reconstruction produced SSIM 0.99, CNR 16.8, SNR 29.6, TIW-D 1.4 and TIW-T 1.4 compared with variable angular gap 30° with the MCMKB reconstruction which had SSIM 0.98, CNR 11.3, SNR 37.9, TIW-D 1.7 mm and TIW-T 1.9 mm. CNR, SNR, and TIW metrics were mostly impacted by the increase in variable angular gap. However, the qualitative analysis showed that the images appear to be very similar regardless of the variable angular gap size. These findings (CNR, SNR, TIW-D, and TIW-T) were also observed in FDK and MKB reconstructions with the exception of SSIM. In the absence of motion compensated reconstruction (MCMKB), increasing the variable angular gap consistently deteriorated the SSIM result for FDK and MKB reconstructions.

#### 3.2.2 | Static angular gaps

Static angular gaps of 20°, 30°, and 40° had the greatest reduction in visual image quality (Figure 5). All reconstructions exhibited considerably more streaking and noise artifacts than the ideal angular separation reconstructions. FDK reconstructions maintained most of the anatomical features of the XCAT phantom but with considerable streaking artifacts. This had negative downstream effects on MKB reconstructions, where anatomical features, especially around the heart region, were greatly distorted. Streak and noise artifacts increased as the static angular gap increased in size. MCMKB reconstructions were able to reduce a large proportion of streaking and noise artifacts but were still more prevalent than ideal angular separation reconstructions. At a static angular gap of 30°, MCMKB reconstruction had difficulty maintaining sharp edges around the heart, and at 40°, anatomical features could not be resolved. Nevertheless, the static angular gap 20° MCMKB reconstruction was the most visually similar to the ideal angular separation MCMKB reconstruction. Static angular gap reconstructions between 20° and 30° appeared to be the upper limit before the visual image quality of the reconstructions started to degrade or deviate from ideal angular separation reconstructions.

Static angular gaps of 30° and 40° with FDK, MKB, and MCMKB reconstructions showed the largest reduction in image quality metric results (Tables 2–6). Static angular gaps of 30° and 40°FDK, MKB, and MCMKB reconstructions generally produced image quality metric values less than ideal angular separation reconstructions. For example, the static angular gap 40° MCMKB reconstructions produced SSIM 0.92, CNR 6.8, SNR 6.7, TIS-D 5.7 mm, and TIW-T 5.9 mm compared to ideal angular separation MCMKB reconstruction of SSIM 1.00, CNR 13.6, SNR 34.8, TIW-D 1.5 mm and TIW-T 2.0 mm. Additionally, FDK and MKB reconstructions were most severely impacted as TIW-D and TIW-T metrics did not converge to a result and SSIM consistently deteriorated when the static angular gap was increased. MCMKB reconstructions were able to improve the image quality metric results for static angular gap 30° and 40° reconstructions, but results were still much lower than the ideal angular separation MCMKB reconstructions. Of all the static angular gap sizes only the 20° gap with FDK, MKB, and MCMKB reconstructions produced similar CNR, SNR, TIW-D, and TIW-T results compared to the ideal angular separation reconstructions. The static angular gap 20° MCMKB reconstructions produced SSIM 0.99, CNR 17.4, SNR 24.7, TIS-D 1.5 mm, and TIW-T 2.3 while ideal angular separation MCMKB reconstructions produced SSIM 1.00, CNR 13.6, SNR 34.8, TIS-D 1.5 mm and TIW-T 2.0 mm. Increasing the size of the static angular gap resulted in reductions across all image quality metrics. Based on the image quality metric results, a static angular gap of 20°–30° appeared to be the upper limit before reconstructions produced lower image quality metric values than the ideal angular separation reconstructions.

### 3.3 | The effect of adaptive velocity acquisition on image quality

**FIGURE 6:**
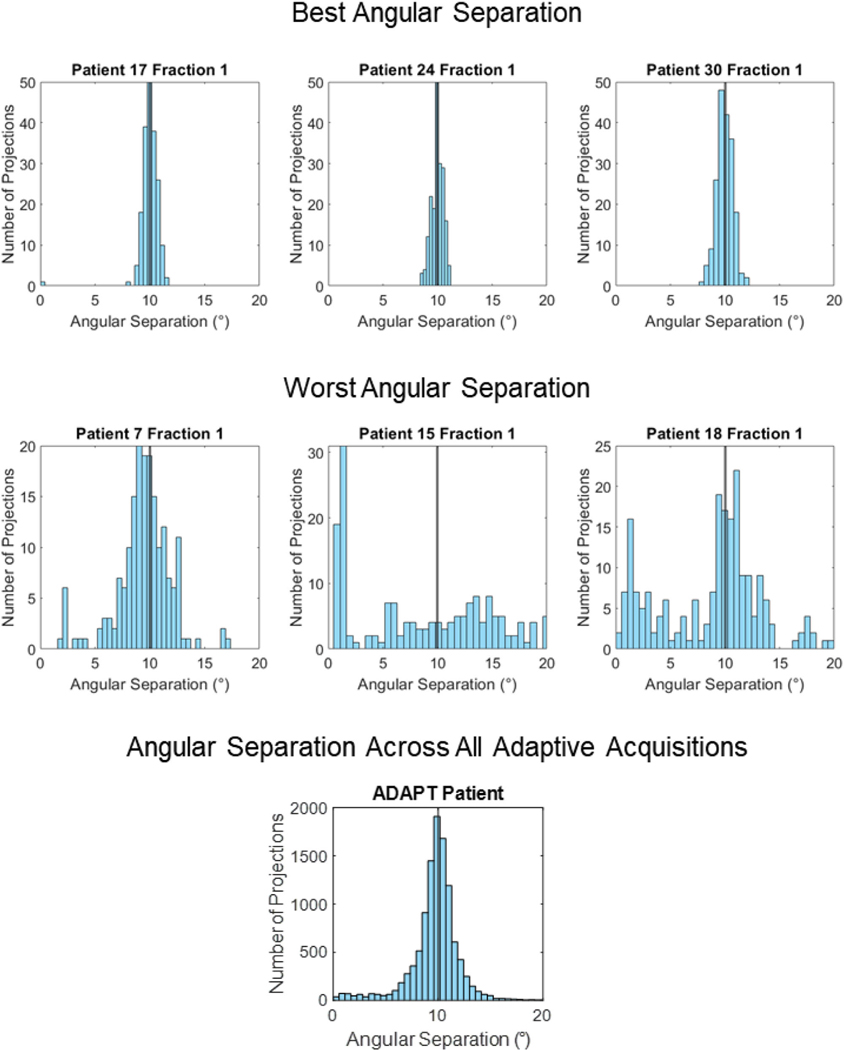
Histograms of the best, worst and all angular separation between x-ray projections observed in the ADAPT clinical trial. Black line represents even 10° angular spacing, the ideal angular separation between x-ray projections.

Figure 6 provides histograms of the angular separation between x-ray projections from the ADAPT clinical trial. Figure 5 provides image reconstructions of the simulated acquisitions using breathing data from the ADAPT clinical trial.

The data from the ADAPT clinical trial achieved an average angular separation of 9.95°± 0.07°, compared to the ideal angular separation of 10° and a relatively small angular RMSE 1.72°± 1.17° (Table 1 and Appendix Table SA1). However, when examining individual patient scans there were some scans where the maximum angular separation was distinctly different to the ideal angular separation. Namely fraction 1 of patients 7, 15, and 18, all of which incurred the largest maximum angular separation (more than 20°) and worst angular RMSE (more than 3) compared to the ideal due to patients holding their breath (patient 7) or difficulties in the system calculating the respiratory phase in real-time (patient 15 and 18). Angular separation histograms (Figure 6) show that fraction 1 of patients 7, 15, and 18 had inconsistent angular separation and clearly did not resemble the ideal. All three of these acquisitions contained static angular gaps of approximately 20°−30°. These were scans with the worst angular separation from the ADAPT clinical trial. In contrast, the best performing scans in terms of angular separation, fraction 1 of patients 17, 24 and 30 saw a narrow distribution of projections around the 10° region closely resembling ideal angular separation (Figure 6). These scans also had the lowest angular RMSE, and maximum angular separation of patient scans compared to ideal angular separation. The rest of the patient scans also had narrow histograms around the 10° region resembling the best angular separation scans of the ADAPT clinical trial.

#### 3.3.1 | Qualitative analysis (adaptive velocity acquisition)

**FIGURE 7:**
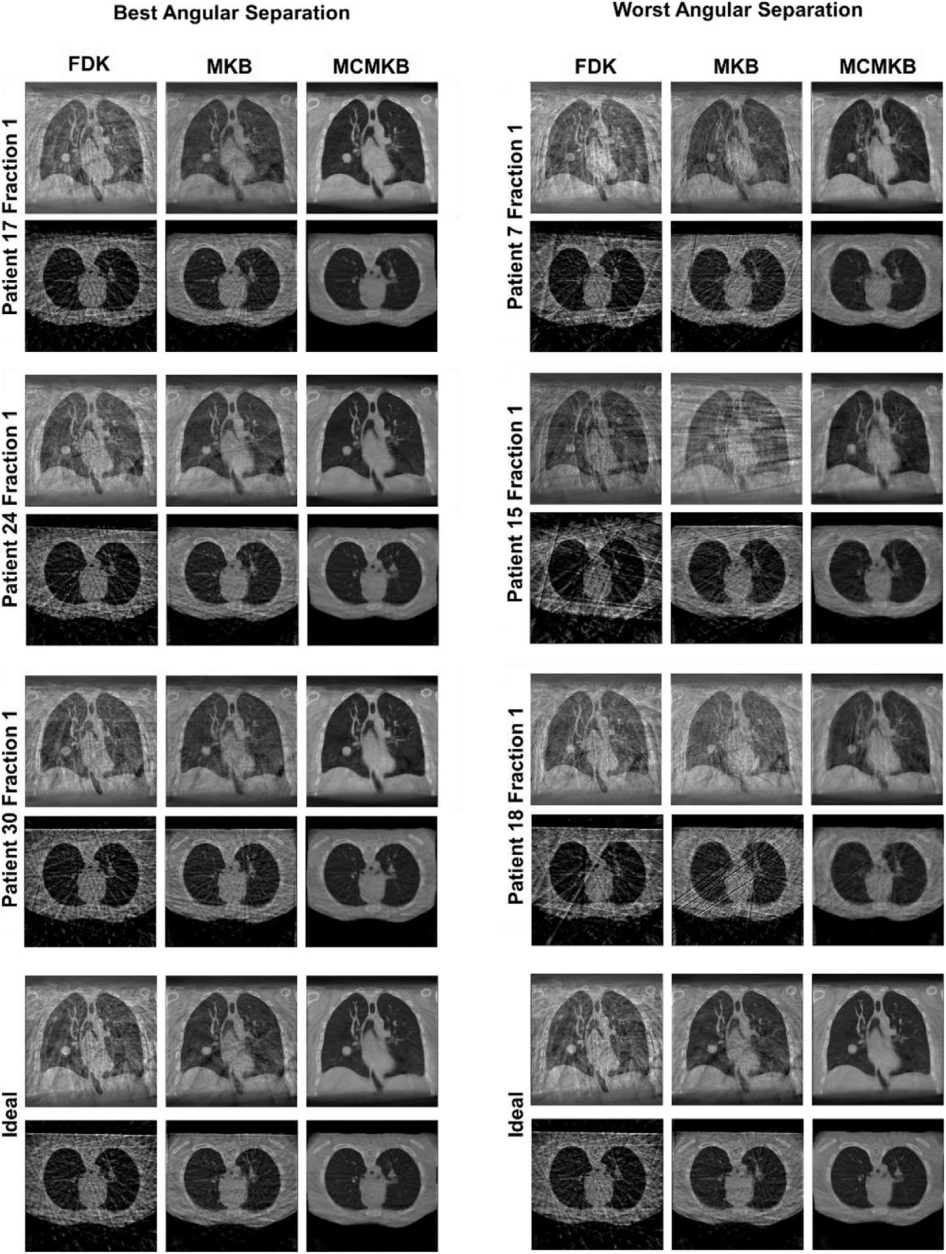
Reconstruction images for the best and worst angular separation observed in the ADAPT clinical trial with ideal angular separation. Ideal angle separation image reconstructions are included for reference. FDK, MKB, and MCMKB reconstructions at peak exhale phase 1 are displayed.

Overall, the image quality of FDK, MKB, and MCMKB reconstructions using the ADAPT clinical trial patients acquired angles closely resembled to the ideal angular separation reconstructions (Figure 7). However, patients with an angular spread that deviated from the ideal led to reduced visual image quality such as fraction 1 of patients 7, 15, and 18. Image quality for fraction 1 of patients 7, 15, and 18 were considerably streakier and noisier than the ideal separation reconstructions across all image reconstruction methods (Figure 7). FDK and MKB reconstructions had more difficulty in resolving anatomical features, for example for fraction 1 of patient 15 the heart and tumor region was greatly distorted by the streaking artifacts (Figure 7). The MCMKB-reconstructions for fraction 1 of patients 7, 15, and 18 resolved most of the streaking artifacts and noise encountered in the FDK and MKB reconstructions, however, the visual image quality was still worse than the ideal angular separation reconstructions. The main contributor to the reduction in image quality was the presence of static angular gaps of 20°–30° in these acquisitions as no projection data was acquired for these regions. These patients were likely holding their breath for a period of time during imaging, resulting in these static angular gaps (patient 7) or there was an error reading the respiratory signal for an extended period of time (patients 15 and 18).

#### 3.3.2 | Quantitative analysis (adaptive velocity acquisition)

In general, ADAPT clinical trial patient projection angles produced image quality metrics results similar to the ideal angular separation for FDK, MKB, and MCMKB reconstructions (Tables 2–6). The qualitative analysis demonstrated worst angular separation cases (caused by static angular gaps) resulted in a substantial reduction in visual image quality and the same effect was observed in the quantitative metrics. For example, patient 15 fraction 1 with the MCMKB reconstruction had SSIM 0.97, CNR 8.1, SNR 14.7, TIW-D 1.9 mm, and TIW-T 2.8 mm compared to ideal angular separation MCMKB reconstruction of SSIM 1.00, CNR 13.6, SNR 34.8, TIW-D 1.5 mm, and TIW-T 2.0 mm. The best angular separation cases resulted in improved image quality metric results and were similar to the ideal angular separation MCMKB reconstructions. An example is patient 24 fraction 1 MCMKB reconstruction which produced SSIM 0.99, CNR 12.8, SNR 36.6, TIW-D 1.3 mm, and TIW-T 2.3 mm. These findings (CNR, SNR, TIW-D, and TIW-T) were also observed in FDK and MKB reconstructions with the exception of SSIM. SSIM was most impacted for FDK (SSIM 0.84 ± 0.02), and MKB (SSIM 0.92 ± 0.02) reconstructions compared to the ideal angular separation reconstructions. The implication is that without motion-compensated reconstruction (MCMKB), SSIM can deteriorate when angular separation deviates from ideal angular separation.

## 4 | DISCUSSION

With the emergence of fast-rotating gantries, there is now the opportunity to acquire fast-rotation 4DCBCT images for patient positioning. This study examined the impact that fast gantry rotation has on the resulting image quality, including the current clinical standard using a constant gantry rotation speed, as well as variable and static angular gaps that can result due to breath hold scenarios. Additionally, a method that adapts gantry rotation speed to the patient’s breathing was also investigated. The XCAT phantom was selected for projection simulation to remove patient-specific image quality variables. While producing simulated projections using real patient data may offer an additional dimension of analysis, this study focused on isolating the effect of projection location and examining the distribution of projections in practice. Our results also provide useful information for future clinical implementations of 4DCBCT imaging, particularly if the scan time is shortened from the standard 4-min acquisition that is often used in clinical practice.

Currently, achieving adaptive 4DCBCT in clinical settings would require substantial hardware modifications if highly precise gantry control is needed to achieve the ideal gantry angle separation. To investigate this, the first part of the study investigated the impact of constant velocity acquisition and image reconstruction method on image quality which represents as simple methods to implement on fast rotating linear accelerators without significant hardware upgrades. The qualitative and quantitative results showed FDK, MKB, and MCMKB reconstructions struggled to maintain the image quality of ideal reconstructions regardless of the gantry speed of the constant velocity acquisitions. The fastest gantry speeds 9.2 s (21°/s) and 60 s (3.3°/s), suffered the greatest reduction in image quality (SSIM, SNR, CNR, TIW-D, and TIW-T) with substantial motion blurring and streaking for all reconstruction methods. Slowing the gantry speed down to 120 s (1.6°/s) or 240 s (0.8°/s) improved the image quality (visually, SNR and CNR) however, the diverse angular gaps and uneven projection binning were still prominent. It was common for some respiratory phases to have substantially fewer than the ideal 20 projections (as low as 2 projections), which led to poor image quality FDK and MKB reconstructions. As a result, motion and streaking artifacts propagated into the motion-compensated reconstructions (MCMKB) because accurate DVFs could not be estimated from FDK and MKB volumes that were produced from undersampled projections. These findings highlight the rationale for an adaptive approach to overcome data insufficiency in certain respiratory phases for fast low-dose 4DCBCT.

Both variable and static angular separation was observed in the adaptive and constant velocity simulation, posing a potential risk to image quality. Variable angular separation occurs when the patient has rapidly changing and irregular breathing patterns, causing the gantry to miss a respiratory bin. In contrast, static angular separations occur when the patient pauses breathing for an extended period, leading to data not being acquired for a particular angular range. These studies identified the angular separation conditions in fast low-dose acquisitions that impair image quality. The results of these simulations will guide vendors in developing solutions to address issues such as completely stopping the gantry and ensuring the relevant projections are acquired for each respiratory bin. Furthermore, these simulations emphasize the importance of motion-compensated reconstructions, as image quality was improved in all simulated scenarios regardless of the type of gantry used and the severity of the angular separation.

The qualitative and quantitative results showed that the presence of variable angular gaps (20°, 30°, and 40°) in MCMKB reconstructions maintained the image quality of ideal angular separation reconstructions. For FDK and MKB reconstructions, while the majority of the image quality metrics (SNR, CNR, TIW-D, TIW-T) were similar to the ideal angular reconstructions, SSIM was most impacted for variable angular gaps. The implication is that FDK and MKB reconstruction algorithms are sensitive to small to moderate deviations in angular separation. Static angular gaps (30° and 40°) demonstrated the greatest reductions to visual and image quality metric results. Static angular gap (30° and 40°) FDK and MKB reconstructions were high in streaking and noise artifacts, motion compensated reconstruction (MCMKB) was able to reduce the severity of these artifacts, but the image quality was still worse than the ideal angular separation reconstructions. Like the worst angular separation cases, the diaphragm and the tumor regions were greatly affected by the loss of sharpness and contrast in the diaphragm and tumor regions (reduced SSIM, CNR, TIW-D, and TIW-T). Among all the static angular gap cases, only the static angular gap 20° reconstructions (FDK, MKB, and MCMKB) were able to maintain the visual and image quality metric results of the ideal angle separation reconstructions.

This is the first study to investigate the impact of angular separation between projections on adaptive acquisition. Previous adaptive 4DCBCT studies have demonstrated promising results in reducing imaging scan time and dose while producing image quality comparable to clinical standards. However, it is challenging to predict whether adaptive acquisition in clinical practice can achieve precise gantry control and its impact on image quality. The results showed that adaptive acquisition performed well in the ADAPT clinical trial by ensuring approximately the same number of projections in each respiratory bin and relatively even angular separation between projections.

The adaptive 4DCBCT approach attempts to compensate for large angular gaps by adapting the machine to the patient’s breathing. Therefore, the final part of this study focused on analyzing the angular separation that exists in the ADAPT patient clinical trial and their impact on reconstruction performance. Patient projection acquisitions achieved an average angular separation of 9.95° ± 0.10°, maximum angular separation of 14.80 ± 3.15 and angular RMSE of 1.72 ± 1.17, closely resembling ideal angular separation. However, during the clinical trial, we observed a small number of occasions where the angular separation deviated from the mean, which occur with the worst angular separation cases because we were unable to completely stop the gantry during large breathing pauses or due to challenges in the ADAPT algorithms computing the real-time phase. These are problems that could be fixed with vendor support. The deviations had downstream impacts on reconstruction performance (FDK, MKB, and MCMKB) with reductions to visual image quality and to all image quality metrics compared to the ideal angular separation reconstructions. Specifically, these reconstructions were affected by increased streaking and noise artifacts which led to a loss in contrast and sharpness at the diaphragm and tumor regions (reduced SSIM, CNR, TIW-D, and TIW-T).

Our analysis indicates that image quality starts to degrade with an angular gap of 20° or larger. Motion compensated (MCMKB) reconstruction using the ADAPT patient projection angles possessed visual and image quality metric results similar to the ideal angular separation reconstructions, indicating that the reconstruction algorithm was able to compensate for small to moderate deviations in angular separation.

Several conclusions may be drawn from these results. This study demonstrates that acquiring fast low-dose 4DCBCT acquisitions (200 projections) using a constant velocity gantry is not a feasible option for producing reconstructions that are similar to the ideal angular separation 4DCBCT reconstructions. The main culprit is the uneven projection sampling on a constant velocity acquisition, causing images to be distorted in undersampled respiratory phases. Even when motion compensated reconstruction (MCMKB) was employed to utilize all the projection data available to generate each phase, motion and streaking artifacts still propagated throughout all volumes. Moreover, methods such as iterative reconstruction may struggle with the same issue, as there is simply insufficient data in certain respiratory phases to compute accurate DVFs. This is also the reason why conventional 4DCBCT acquisitions are highly sampled (1320 projections) to ensure all respiratory phases can generate images with accurate anatomy.

The results identified a static angular gap between 20°–30° is the upper limit before reconstruction image quality becomes worse than ideal angular separation reconstructions (FDK, MKB, and MCMKB). As the static angular gap increases beyond the limit, FDK and MKB reconstructions image quality metrics (SSIM, CNR, SNR, TIW-D, and TIW-T) degrade rapidly, and MCMKB reconstructions are no longer qualitatively and quantitatively similar to the ideal angular separation MCMKB reconstructions.

Precise angular spacing during fast low-dose adaptive 4DCBCT acquisition may not be necessary to achieve the image quality of ideal angular separation reconstructions in the context of patient positioning in radiotherapy. This was demonstrated with a variable angular gap (20°, 30°, and 40°) motion-compensated reconstructions (MCMKB), which were able to maintain the image quality of ideal angular separation reconstructions. This also suggests acquiring a sufficient number of projections in each respiratory phase across the entire 200° arc has a larger influence on reconstruction image quality than the precise angular location of projections. However, for applications of 4DCBCT requiring higher precision than that needed for patient positioning, more precise gantry control may be needed.

Last, for fast low-dose adaptive 4DCBCT, motion compensation reconstruction (MCMKB), and potentially iterative methods, may be crucial to produce images with improved contrast, sharpness, and reduced noise (as opposed to FDK or MKB reconstructions). As fast low-dose 4DCBCT acquisitions have a low projection count, streaking and noise artifacts are inevitable and the MCMKB method combats this by ensuring all projections are utilized to generate the final respiratory binned volumes.

To maintain the clinical benefit of scan time reduction, reconstruction algorithm selection was restricted to those having the potential to be computed fast enough to suit patient treatment fraction timeframes. Our implementations of FDK, MKB, and MCMKB required approximately 1, 1, and 7 min on our hardware respectively. Currently, the most time-consuming step of MCMKB reconstruction is DVF estimation and our unoptimized implementation generates each respiratory phase in series.

A limitation of this study was the exclusion of iterative reconstruction methods such as SMEIR^8^ (Simultaneous Motion Estimation and Image Reconstruction), MC-SART^17^ (Motion Compensated Simultaneous algebraic reconstruction technique), and ROOSTER.^38^ While these methods may improve reconstruction image quality for fast low-dose adaptive 4DCBCT, reconstructions are computationally expensive (over 1.5 h on our hardware), which may not be suitable for current clinical timeframes where the images are used for patient positioning. Filtered back projection algorithms are known to perform poorly during limited and uneven angular sampling, however, in this study, these algorithms performed well in most simulated angular scenarios, suggesting angular separation will need to be much more severe before the benefit of iterative reconstruction outweighs its computational cost.

The adaptive 4DCBCT approach is observed to extend the scan time compared to the Halcyon 9.2-s scan. While a typical adaptive 4DCBCT scan requires 60−80 s, it is still considered a very fast 4DCBCT scan, as it represents a reduction of up to 75% in scan time compared to the clinical 4DCBCT scan, which takes 4 min. Currently, the Halcyon system employs a 3D acquisition protocol, provided that motion-compensated reconstruction performs well with limited data, this study explored the possibility of 4D acquisitions on the fast-rotating gantries. Moreover, it should be noted that the Halcyon 9.2-s scan covers approximately 3−4 respiratory cycles, which may not sufficiently represent a patient’s breathing pattern. Therefore, in addition to the 9.2-s acquisition, this study investigated adaptive acquisition, as determining the number of respiratory cycles to sample remains an ongoing clinical question.

Overall, these simulation studies offer important knowledge regarding the impact of gantry velocity and angular separation on image reconstruction. This knowledge will be useful for future hardware and protocol development for fast low-dose 4DCBCT acquisition systems. The results demonstrate adaptive velocity gantry systems in conjunction with motion-compensated reconstruction may be a good option for fast low-dose 4DCBCT (200-projections) to make full use of the emerging generation of faster rotating linear accelerators. For fast constant rotating systems, (18°–22° per second) such as the Varian Halcyon,^39^ Accuray Radixact,^40^ and Elekta Versa HD^41^ will require more projections to match the image quality of the ideal reconstructions presented. However, this conflicts with the ALARA principle and eventually result in the same drawbacks of conventional 4DCBCT (streaking artifacts and high imaging dose).

## 5 | CONCLUSION

With the emergence of fast gantry rotation systems very fast 4DCBCT scans can be acquired provided that the entire scan range is adaptively sampled, and motion-compensated reconstruction is performed in the context of patient positioning in radiotherapy.

## Supplementary Material

Table SA1

## Figures and Tables

**TABLE 1:** Angular separation between projections of simulated and patient acquired scans.

Projections	Acquisition	Average angular separation (°)	Maximum angular separation (°)	Angular RMSE
200	Ideal Angular Separation	10.00	10.00	N/A
200	Constant Velocity 9.2 s	6.82 ± 18.14	61.02 ± 25.87	19.58 ± 7.37
200	Constant Velocity 60 s	9.37 ± 9.53	31.49 ± 15.44	9.87 ± 6.14
200	Constant Velocity 120 s	9.39 ± 9.38	32.93 ± 14.46	10.03 ± 5.82
200	Constant Velocity 240 s	9.27 ± 9.78	33.97 ± 15.42	10.62 ± 6.88
200	Variable Angular Gap 20°	9.69	22.19	2.58
200	Variable Angular Gap 30°	9.57	32.43	4.93
200	Variable Angular Gap 40°	9.61	40.85	7.07
200	Static Angular Gap 20°	10.46	20.52	2.28
200	Static Angular Gap 30°	10.99	30.52	4.51
200	Static Angular Gap 40°	11.51	40.52	6.78
200	ADAPT Patient Mean	9.95 ± 0.10	14.80 ± 3.15	1.72 ± 1.17

**TABLE 2:** Structural similarity index.

Structural SIMilarity (ideal reference, higher is better)			

	FDK	MKB	MCMKB
Ideal	1.00	1.00	1.00
Constant Velocity 9.2 s	0.78 ± 0.02	0.94 ± 0.01	0.97 ± 0.00
Constant Velocity 60 s	0.83 ± 0.02	0.95 ± 0.01	0.98 ± 0.00
Constant Velocity 120 s	0.85 ± 0.01	0.95 ± 0.00	0.98 ± 0.00
Constant Velocity 240 s	0.84 ± 0.01	0.95 ± 0.01	0.98 ± 0.01
Variable Gap 20°	0.84	0.96	0.99
Variable Gap 30°	0.83	0.93	0.99
Variable Gap 40°	0.82	0.92	0.99
Static Gap 20°	0.92	0.94	0.99
Static Gap 30°	0.89	0.90	0.96
Static Gap 40°	0.88	0.88	0.92
ADAPT Patient Mean	0.84 ± 0.02	0.92 ± 0.02	0.98 ± 0.01

**TABLE 3:** Contrast to noise ratio.

Contrast to noise ratio (higher is better)			

	FDK	MKB	MCMKB
Ideal	4.2	6.9	13.6
Constant Velocity 9.2 s	2.3 ± 0.37	11.9 ± 3.2	13.1 ± 3.5
Constant Velocity 60 s	2.9 ± 0.4	9.9 ± 1.6	12.6 ± 1.7
Constant Velocity 120 s	3.6 ± 0.3	10.2 ± 1.8	16.0 ± 2.7
Constant Velocity 240 s	3.7 ± 0.4	11.2 ± 2.4	16.7 ± 2.2
Variable Gap 20°	4.0	7.2	16.8
Variable Gap 30°	4.2	6.7	11.3
Variable Gap 40°	3.7	5.6	13.7
Static Gap 20°	3.5	6.0	17.4
Static Gap 30°	3.0	4.1	11.0
Static Gap 40°	3.5	3.3	6.8
ADAPT Patient Mean	4.0 ± 0.3	6.2 ± 1.2	12.4 ± 2.0

**TABLE 4:** Signal to noise ratio.

Signal to noise ratio (higher is better)			

	FDK	MKB	MCMKB
Ideal	6.7	10.0	34.8
Constant Velocity 9.2 s	4.6 ± 0.9	33.5 ± 10.3	35.4 ± 12.7
Constant Velocity 60 s	5.2 ± 0.7	23.2 ± 6.3	39.6 ± 8.4
Constant Velocity 120 s	5.8 ± 0.63	19.4 ± 3.2	42.7 ± 10.4
Constant Velocity 240 s	6.0 ± 0.7	22.6 ± 5.1	43.9 ± 10.9
Variable Gap 20°	6.8	10.9	29.6
Variable Gap 30°	6.4	10.0	37.9
Variable Gap 40°	5.3	8.5	34.7
Static Gap 20°	6.9	7.6	24.7
Static Gap 30°	5.6	5.7	21.6
Static Gap 40°	7.1	4.1	6.7
ADAPT Patient Mean	6.3 ± 0.6	8.7 ± 1.1	28.8 ± 6.7

**TABLE 5:** Tissue interface width—diaphragm.

Tissue interface width diaphragm (mm, lower is better)			

	FDK	MKB	MCMKB
Ideal	3.1	2.4	1.5
Constant Velocity 9.2 s	6.6 ± 7.1	6.1 ± 12.2	5.8 ± 12.3
Constant Velocity 60 s	4.6 ± 21.8	4.3 ± 15.3	4.4 ± 6.5
Constant Velocity 120 s	3.3 ± 15.4	3.2 ± 28.9	3.9 ± 10.4
Constant Velocity 240 s	3.2 ± 7.6	3.3 ± 22.4	4.1 ± 8.6
Variable Gap 20°	2.6	2.4	1.4
Variable Gap 30°	3.1	2.9	1.7
Variable Gap 40°	4.1	3.3	1.8
Static Gap 20°	3.1	3.0	1.5
Static Gap 30°	–	–	1.9
Static Gap 40°	–	–	5.7
ADAPT Patient Mean	2.4 ± 0.3	2.2 ± 0.6	1.2 ± 0.1

**TABLE 6:** Tissue interface width—tumor.

Tissue interface width tumor (mm, lower is better)			

	FDK	MKB	MCMKB
Ideal	2.7	2.8	2.0
Constant Velocity 9.2 s	1.2 ± 0.36	5.3 ± 8.1	5.3 ± 12.1
Constant Velocity 60 s	5.6 ± 18.8	5.0 ± 23.8	4.5 ± 15.4
Constant Velocity 120 s	3.6 ± 14.9	2.9 ± 17.0	3.5 ± 14.0
Constant Velocity 240 s	3.5 ± 10.8	2.9 ± 14.3	3.6 ± 9.1
Variable Gap 20°	2.1	2.2	1.4
Variable Gap 30°	3.4	2.5	1.9
Variable Gap 40°	4.4	3.3	1.8
Static Gap 20°	2.4	4.0	2.3
Static Gap 30°	3.1	–	3.5
Static Gap 40°	5.2	–	5.9
ADAPT Patient Mean	2.9 ± 0.8	3.7 ± 0.9	2.4 ± 0.3
