# Supplementary material for: Faster and lower dose imaging: evaluating adaptive, constant gantry velocity and angular separation in fast low-dose 4D cone beam CT imaging
Source: Med Phys. Author manuscript; Available in PMC 2024 Nov 1. (PMC11528889; doi:10.1002/mp.16585)
Supplement: Table SA1 [file NIHMS1939933-supplement-Table_SA1.docx]

Table 1. Angular separation between projections of simulated and patient acquired scans.

| **Projections** | **Acquisition** | **Average angular separation (°)** | **Maximum angular separation (°)** | **Angular RMSE** |
| --- | --- | --- | --- | --- |
| 200 | Ideal Angular Separation | 10.00 | 10.00 | N/A |
| 200 | Constant Velocity 9.2s | 6.82 ± 18.14 | 61.02 ± 25.87 | 19.58 ± 7.37 |
| 200 | Constant Velocity 60s | 9.37 ± 9.53 | 31.49 ± 15.44 | 9.87 ± 6.14 |
| 200 | Constant Velocity 120s | 9.39 ± 9.38 | 32.93 ± 14.46 | 10.03 ± 5.82 |
| 200 | Constant Velocity 240s | 9.27 ± 9.78 | 33.97 ± 15.42 | 10.62 ± 6.88 |
| 200 | Variable Angular Gap 20° | 9.69 | 22.19 | 2.58 |
| 200 | Variable Angular Gap 30° | 9.57 | 32.43 | 4.93 |
| 200 | Variable Angular Gap 40° | 9.61 | 40.85 | 7.07 |
| 200 | Static Angular Gap 20° | 10.46 | 20.52 | 2.28 |
| 200 | Static Angular Gap 30° | 10.99 | 30.52 | 4.51 |
| 200 | Static Angular Gap 40° | 11.51 | 40.52 | 6.78 |
| 200 | ADAPT Patient Mean | 9.95 ± 0.10 | 14.80 ± 3.15 | 1.72 ± 1.17 |

Table 2: Structural Similarity Index

| Structural SIMilarity (Ideal Reference, Higher is Better) | | | |
| --- | --- | --- | --- |
|  | FDK | MKB | MCMKB |
| Ideal | 1.00 | 1.00 | 1.00 |
| Constant Velocity 9.2s | 0.78 ± 0.02 | 0.94 ± 0.01 | 0.97 ± 0.00 |
| Constant Velocity 60s | 0.83 ± 0.02 | 0.95 ± 0.01 | 0.98 ± 0.00 |
| Constant Velocity 120s | 0.85 ± 0.01 | 0.95 ± 0.00 | 0.98 ± 0.00 |
| Constant Velocity 240s | 0.84 ± 0.01 | 0.95 ± 0.01 | 0.98 ± 0.01 |
| Variable Gap 20° | 0.84 | 0.96 | 0.99 |
| Variable Gap 30° | 0.83 | 0.93 | 0.99 |
| Variable Gap 40° | 0.82 | 0.92 | 0.99 |
| Static Gap 20° | 0.92 | 0.94 | 0.99 |
| Static Gap 30° | 0.89 | 0.90 | 0.96 |
| Static Gap 40° | 0.88 | 0.88 | 0.92 |
| ADAPT Patient Mean | 0.84 ± 0.02 | 0.92 ± 0.02 | 0.98 ± 0.01 |

Table 3: Contrast to Noise Ratio

| Contrast to Noise Ratio (Higher is Better) | | | |
| --- | --- | --- | --- |
|  | FDK | MKB | MCMKB |
| Ideal | 4.2 | 6.9 | 13.6 |
| Constant Velocity 9.2s | 2.3 ± 0.37 | 11.9 ± 3.2 | 13.1 ± 3.5 |
| Constant Velocity 60s | 2.9 ± 0.4 | 9.9 ± 1.6 | 12.6 ± 1.7 |
| Constant Velocity 120s | 3.6 ± 0.3 | 10.2 ± 1.8 | 16.0 ± 2.7 |
| Constant Velocity 240s | 3.7 ± 0.4 | 11.2 ± 2.4 | 16.7 ± 2.2 |
| Variable Gap 20° | 4.0 | 7.2 | 16.8 |
| Variable Gap 30° | 4.2 | 6.7 | 11.3 |
| Variable Gap 40° | 3.7 | 5.6 | 13.7 |
| Static Gap 20° | 3.5 | 6.0 | 17.4 |
| Static Gap 30° | 3.0 | 4.1 | 11.0 |
| Static Gap 40° | 3.5 | 3.3 | 6.8 |
| ADAPT Patient Mean | 4.0 ± 0.3 | 6.2 ± 1.2 | 12.4 ± 2.0 |

Table 4: Signal to Noise Ratio

| Signal to Noise Ratio (Higher is Better) | | | |
| --- | --- | --- | --- |
|  | FDK | MKB | MCMKB |
| Ideal | 6.7 | 10.0 | 34.8 |
| Constant Velocity 9.2s | 4.6 ± 0.9 | 33.5 ± 10.3 | 35.4 ± 12.7 |
| Constant Velocity 60s | 5.2 ± 0.7 | 23.2 ± 6.3 | 39.6 ± 8.4 |
| Constant Velocity 120s | 5.8 ± 0.63 | 19.4 ± 3.2 | 42.7 ± 10.4 |
| Constant Velocity 240s | 6.0 ± 0.7 | 22.6 ± 5.1 | 43.9 ± 10.9 |
| Variable Gap 20° | 6.8 | 10.9 | 29.6 |
| Variable Gap 30° | 6.4 | 10.0 | 37.9 |
| Variable Gap 40° | 5.3 | 8.5 | 34.7 |
| Static Gap 20° | 6.9 | 7.6 | 24.7 |
| Static Gap 30° | 5.6 | 5.7 | 21.6 |
| Static Gap 40° | 7.1 | 4.1 | 6.7 |
| ADAPT Patient Mean | 6.3 ± 0.6 | 8.7 ± 1.1 | 28.8 ± 6.7 |

Table 5: Tissue Interface Width – Diaphragm

| Tissue Interface Width Diaphragm (mm, Lower is Better) | | | |
| --- | --- | --- | --- |
|  | FDK | MKB | MCMKB |
| Ideal | 3.1 | 2.4 | 1.5 |
| Constant Velocity 9.2s | 6.6 ± 7.1 | 6.1 ± 12.2 | 5.8 ± 12.3 |
| Constant Velocity 60s | 4.6 ± 21.8 | 4.3 ± 15.3 | 4.4 ± 6.5 |
| Constant Velocity 120s | 3.3 ± 15.4 | 3.2 ± 28.9 | 3.9 ± 10.4 |
| Constant Velocity 240s | 3.2 ± 7.6 | 3.3 ± 22.4 | 4.1 ± 8.6 |
| Variable Gap 20° | 2.6 | 2.4 | 1.4 |
| Variable Gap 30° | 3.1 | 2.9 | 1.7 |
| Variable Gap 40° | 4.1 | 3.3 | 1.8 |
| Static Gap 20° | 3.1 | 3.0 | 1.5 |
| Static Gap 30° | - | - | 1.9 |
| Static Gap 40° | - | - | 5.7 |
| ADAPT Patient Mean | 2.4 ± 0.3 | 2.2 ± 0.6 | 1.2 ± 0.1 |

Table 6: Tissue Interface Width – Tumor

| Tissue Interface Width Tumor (mm, Lower is Better) | | | |
| --- | --- | --- | --- |
|  | FDK | MKB | MCMKB |
| Ideal | 2.7 | 2.8 | 2.0 |
| Constant Velocity 9.2s | 1.2 ± 0.36 | 5.3 ± 8.1 | 5.3 ± 12.1 |
| Constant Velocity 60s | 5.6 ± 18.8 | 5.0 ± 23.8 | 4.5 ± 15.4 |
| Constant Velocity 120s | 3.6 ± 14.9 | 2.9 ± 17.0 | 3.5 ± 14.0 |
| Constant Velocity 240s | 3.5 ± 10.8 | 2.9 ± 14.3 | 3.6 ± 9.1 |
| Variable Gap 20° | 2.1 | 2.2 | 1.4 |
| Variable Gap 30° | 3.4 | 2.5 | 1.9 |
| Variable Gap 40° | 4.4 | 3.3 | 1.8 |
| Static Gap 20° | 2.4 | 4.0 | 2.3 |
| Static Gap 30° | 3.1 | - | 3.5 |
| Static Gap 40° | 5.2 | - | 5.9 |
| ADAPT Patient Mean | 2.9 ± 0.8 | 3.7 ± 0.9 | 2.4 ± 0.3 |

# Appendix

Table A1. Angular separation between projections of the fast low-dose adaptive 4DCBCT acquisitions from the ADAPT clinical trial.

|  | **Fraction 1** | | | | **Fraction 2** | | | |
| --- | --- | --- | --- | --- | --- | --- | --- | --- |
| **Patient** | **Proj.** | **Avg. Angular Sep. (°)** | **Max Sep. (°)** | **Angular RMSE** | **Proj.** | **Avg. Angular Sep. (°)** | **Max Sep. (°)** | **Angular RMSE** |
| **1** | 200 | 9.94 | 15.16 | 1.95 | 202 | 9.99 | 15.84 | 1.64 |
| **2** | - | - | - | - | 203 | 10.00 | 14.95 | 1.76 |
| **3** | 200 | 10.01 | 12.91 | 0.88 | 200 | 10.00 | 12.43 | 0.76 |
| **4** | 201 | 9.90 | 16.59 | 1.73 | 202 | 9.70 | 16.95 | 2.98 |
| **5** | 201 | 9.96 | 12.15 | 0.91 | 200 | 10.06 | 13.04 | 1.05 |
| **6** | 200 | 10.00 | 14.83 | 1.54 | 200 | 9.93 | 14.65 | 1.84 |
| **7** | 201 | 9.90 | 24.09 | 3.34 | 202 | 9.84 | 14.33 | 1.95 |
| **8** | 201 | 9.98 | 13.07 | 0.92 | 201 | 10.01 | 12.70 | 1.06 |
| **9** | 202 | 9.93 | 15.95 | 1.71 | - | - | - | - |
| **10** | 201 | 10.03 | 16.98 | 2.17 | 201 | 9.91 | 13.41 | 1.70 |
| **11** | - | - | - | - | 202 | 10.01 | 16.71 | 1.73 |
| **12** | 201 | 9.98 | 12.24 | 0.84 | - | - | - | - |
| **13** | 200 | 9.98 | 13.91 | 1.22 | 200 | 10.00 | 13.96 | 1.58 |
| **14** | 201 | 9.98 | 14.05 | 1.63 | 202 | 9.82 | 13.44 | 1.09 |
| **15** | 200 | 10.26 | 29.04 | 7.58 | 200 | 10.05 | 13.14 | 1.38 |
| **16** | 202 | 9.79 | 13.71 | 1.76 | 201 | 10.00 | 14.52 | 1.87 |
| **17** | 200 | 9.97 | 11.62 | 0.73 | - | - | - | - |
| **18** | 230 | 9.46 | 26.53 | 5.76 | 200 | 10.00 | 15.88 | 1.62 |
| **19** | 202 | 9.96 | 14.81 | 2.01 | 201 | 9.99 | 16.13 | 1.79 |
| **20** | 201 | 10.04 | 14.67 | 2.33 | - | - | - | - |
| **21** | 202 | 9.91 | 16.61 | 2.64 | 204 | 9.97 | 17.53 | 3.19 |
| **22** | - | - | - | - | 200 | 10.00 | 15.91 | 1.95 |
| **23** | 201 | 9.92 | 14.21 | 1.93 | 203 | 9.89 | 12.85 | 1.21 |
| **24** | 200 | 9.97 | 11.19 | 0.52 | 200 | 10.00 | 11.11 | 0.55 |
| **25** | 202 | 10.00 | 13.87 | 1.33 | 201 | 9.96 | 12.10 | 0.77 |
| **26** | 202 | 9.92 | 15.81 | 1.95 | 201 | 9.97 | 14.59 | 2.31 |
| **27** | 200 | 10.04 | 13.18 | 1.39 | 201 | 10.02 | 13.28 | 0.89 |
| **28** | 201 | 10.02 | 13.20 | 1.28 | 201 | 10.00 | 12.50 | 0.67 |
| **29** | 201 | 9.93 | 13.35 | 1.12 | 203 | 9.93 | 12.31 | 0.96 |
| **30** | 200 | 9.97 | 12.12 | 0.74 | 201 | 9.94 | 14.43 | 1.57 |
